# Improvement of the Mass-Rearing Protocols for the South American Fruit Fly for Application of the Sterile Insect Technique

**DOI:** 10.3390/insects12070622

**Published:** 2021-07-09

**Authors:** Thiago Mastrangelo, Adalecio Kovaleski, Bruno Maset, Maria de Lourdes Zamboni Costa, Claudio Barros, Luis Anselmo Lopes, Carlos Caceres

**Affiliations:** 1 Center for Nuclear Energy in Agriculture (CENA/USP), Piracicaba 13416-000, São Paulo, Brazil; piaui@cena.usp.br (T.M.); bamaset@usp.br (B.M.); lia@cena.usp.br (M.d.L.Z.C.); lualopes@cena.usp.br (L.A.L.); 2 Empresa Brasileira de Pesquisa Agropecuária (EMBRAPA), Vacaria 95200-000, Rio Grande do Sul, Brazil; adalecio.kovaleski@embrapa.br (A.K.); claudio.barros@embrapa.br (C.B.); 3 Insect Pest Control Laboratory, Joint FAO/IAEA Centre of Nuclear Techniques in Food and Agriculture, A-2444 Seibersdorf, Austria

**Keywords:** Tephritidae, *Anastrepha fraterculus*, egg incubation, adult density, larval diet, mating compatibility

## Abstract

**Simple Summary:**

Significant advances in the domestication and artificial rearing techniques for the South American fruit fly, *Anastrepha fraterculus* (Diptera, Tephritidae), have been achieved since the FAO/IAEA Workshop held in 1996 in Chile. Despite the availability of rearing protocols that allow the production of a high number of flies, they must be optimized to increase insect yields and decrease production costs. In addition, evidence of sexual incompatibility between a long-term mass-reared Brazilian strain and wild populations has been found. To address these issues, this study refined rearing protocols and assessed the suitability of a bisexual *A. fraterculus* strain established from a target population in southern Brazil for the mass production of sterile flies.

**Abstract:**

The existing rearing protocols for *Anastrepha fraterculus* must be reviewed to make economically viable the production of sterile flies for their area-wide application. Additionally, evidence of sexual incompatibility between a long-term mass-reared Brazilian strain and wild populations has been found. To address these issues, this study aimed to refine rearing protocols and to assess the suitability of an *A. fraterculus* strain for the mass production of sterile flies. A series of bioassays were carried out to evaluate incubation times for eggs in a bubbling bath and to assess the temporal variation of egg production from ovipositing cages at different adult densities. A novel larval diet containing carrageenan was also evaluated. Egg incubation times higher than 48 h in water at 25 °C showed reduced larval and pupal yields. Based on egg production and hatchability, the density of 0.3 flies/cm^2^ can be recommended for adult cages. The diet with carrageenan was suitable for mass production at egg-seeding densities between 1.0 and 1.5 mL of eggs/kg of diet, providing higher insect yields than a corn-based diet from Embrapa. Even after two years of being reared under the new rearing protocols, no sexual isolation was found between the bisexual strain and wild flies.

## 1. Introduction

The genus *Anastrepha* includes more than 300 species of fruit flies, and at least a half dozen of them are pests of native and exotic host plants in Latin America [1,2,3]. The South American fruit fly, *Anastrepha fraterculus*, has primary pest status in several South American countries, including in Brazil [1]. For example, in São Paulo state, *A. fraterculus* is the major fruit fly pest in citrus, and losses in unsprayed orchards can reach up to 25–50% of the total production [4]. The dropping rate of oranges caused by fruit fly species in the 2019–2020 season reached ca. 20.1 million orange boxes (≈USD 92 million), which was the highest since 2015 [5]. In southern Brazil, the fly can cause a reduction in apple and peach production by 30–40% or more [6,7]. In addition, *A. fraterculus* is considered the main pest infesting table grapes [8].

The sterile insect technique (SIT) is an environment-friendly control method that is based on the successive releases of sterile insects over a target area in numbers that overflood the native population and can be used to suppress populations of this major pest [9,10,11]. A program named Moscasul was established in Vacaria, Rio Grande do Sul state in southern Brazil, with the aim to suppress wild *A. fraterculus* populations using sterile flies and parasitoids [12,13]. Among the technical requirements that must be fulfilled before implementing the SIT against this pest, two are of major importance: (1) the availability of mass-rearing technology and (2) the mating compatibility between the reared strain and the target population.

Since the FAO/IAEA Workshop on *A. fraterculus* held in Viña del Mar, Chile, in 1996, significant progress has been made in respect to the domestication and artificial rearing of *A. fraterculus*, resulting in the successful establishment of laboratory colonies in Colombia, Peru, Argentina, and Brazil [14,15,16,17]. Jaldo et al. [15] were the first to propose a potential method for the mass rearing of an Argentinean strain of *A. fraterculus*, which allowed its mass production for 18 generations with a mean egg–pupa recovery of around 44%. Modifications to this initial rearing protocol were introduced by Vera et al. [16], resulting in a substantial increase in egg production (ca. 67 mL eggs/week) and pupal yields (>50,000 pupae/week). In addition, quality control parameters such as larval survival, pupal weight, and adult emergence were improved. In Brazil, Walder et al. [17] showed that the domestication process and quality control parameters of a Brazilian strain originating from infested fruits of a single host (*Eugenia pyriformis* Cambess.) using the proposed artificial rearing system allowed the production of up to 18 L of eggs/generation and >124,000 pupae/week. Despite the rapid expansion of the laboratory colony in Brazil and the production of larger numbers of flies [17], the currently available data suggest that sterile flies from the existing strain (i.e., the *Piracicaba* strain established at the Center for Nuclear Energy in Agriculture (CENA) in 2005) may not be suitable for the control of this pest in southern Brazil [18,19,20].

*Anastrepha fraterculus* is a complex of cryptic species, comprising at least eight morphotypes within its geographical distribution in the Americas [20,21]. In Brazil, three morphotypes have been identified and characterized [22,23], with the Brazilian-1 morphotype predominating in the South and Southeast of the country, whereas the Brazilian-3 morphotype seems to be more prevalent in the coastal plains of the East and North regions, co-occurring with the morphotype 2 in southeastern and northeastern regions [20]. Observed reproductive incompatibilities between the Brazilian-1 morphotype and other morphotypes may reflect the existence of ongoing incipient speciation among *A. fraterculus* populations across their geographical range [24,25,26]. Therefore, the application of the SIT against *A. fraterculus* requires accurate knowledge of the morphotype to ensure that sterility can be induced in the wild target population.

Dias et al. [27] reported full mating compatibility among four populations from southern Brazil. However, partial sexual incompatibility was observed between those populations and the *Piracicaba* strain that had been reared for 10 years (2005–2015) following the procedures of Walder et al. [17]. Such results were not expected since all five populations were identified as members of the same morphotype. The males from the *Piracicaba* strain also mated less frequently with females from two populations originating from the South region than males from the same populations. The loss of sexual compatibility of the *Piracicaba* strain may be due to the continuous laboratory rearing under artificial conditions [28].

Those findings reinforce the importance of selecting a suitable laboratory strain before the release of sterile flies, which must be productive in the rearing facility [28] and also compatible with the native *A. fraterculus* morphotype. Optimizing rearing protocols for a new strain may be essential to avoid colony deterioration due to the rapid accumulation of unfavorable traits (e.g., low tolerance to abiotic stress, fast mating, and reduced courtship) [29,30,31]. Therefore, to know if a new *A. fraterculus* strain originating from the target southern population would be suitable for mass production of sterile flies, this study aimed to answer the following questions: (1) What is the best incubation time for eggs of the strain in a bubbling thermal bath system? (2) What is the temporal variation of egg production from ovipositing cages with different adult densities? (3) What is the suitable egg-seeding density in a larval diet with carrageenan? (4) Of the two larval diets used to rear *A. fraterculus* in Brazil, which one would be best for mass-rearing purposes? (5) After 24 generations under laboratory conditions, would sterile flies of the new strain be compatible with wild flies from the target population?

## 2. Materials and Methods

A new colony from the target population was initiated in late 2015 with wild pupae collected from infested indigenous fruits from the family Myrtaceae, especially from cherry of the Rio Grande (*Eugenia involucrata* DC), guabiroba (*Camponesia xanthocarpa* Berg), and pineapple guava (*Feijoa sellowiana* Berg) in the municipality of Vacaria, Rio Grande do Sul, Brazil (28°31′08″ S, 50°52′18″ W). The parental flies were allowed to oviposit on papaya fruits (*Carica papaya* L.) and on a vertical oviposition panel made of voile cloth coated externally with a thin layer of silicon rubber for 5 generations, following the procedures described by Walder et al. [17]. After a domestication period of 7 generations at the Estação Experimental de Fruticultura de Clima Temperado of the Brazilian Agricultural Research Corporation (Embrapa Grape & Wine, Vacaria), 500 mL of pupae (≈16,500 pupae) were sent to the Food Irradiation and Radioentomology Laboratory of CENA to initiate and expand a colony.

An integrative identification approach [27] confirmed that it was a pure colony of *Anastrepha sp.1 aff. fraterculus* (or the Brazilian-1 morphotype), and it was thereafter named *Vacaria* strain. The colony of this bisexual strain was maintained under controlled environmental conditions (26 ± 2 °C and 70–80% RH) following the rearing protocol of Walder et al. [17] with the following modifications: the use of larger adult cages (75 cm length × 30 cm width × 150 cm height) and a modified larval diet of Salles [32] replacing wheat germ with yellow corn flour and using carrageenan instead of agar (henceforth named CENA’s diet). This larval diet (Table S1) was developed through trial and error in early 2015 for the *Piracicaba* strain, considering the cost and availability of local ingredients (unpublished data). Carrageenan is a gelling agent that can be used in diets for dipterans [33]. Corn flour has relevant texturizing qualities, is low-cost, readily available, and was used successfully to replace wheat germ in a larval diet for *A. fraterculus* [34].

After 12 generations of rearing under CENA’s conditions, the stability of the *Vacaria* strain as assessed by standard quality control parameters (e.g., egg hatch, pupal weight, adult emergence, sex ratio, and flight ability) varied very little among generations (<20%) (Costa, personal communication), and insects from the mother colony started being used for experiments.

### 2.1. Evaluation of the Incubation Time for Eggs in Water

The eggs produced in the rearing colony at CENA were collected every 8 h from the oviposition panels using gentle water sprays that made them drop directly in a black plastic container at the bottom of the cage. Egg collections of each 24 h period were measured volumetrically and transferred to 500 mL Erlenmeyer flasks for air bubbling in filtered water by means of a fish bowl pump (~35 mBar and a density of up to 50 eggs per mL of water). The flasks were then inserted in a thermal bath at 25 °C to assure a more uniform hatching after seeding on the diet. To evaluate different incubation times under such bubbling system, three different periods were tested: 48, 60, and 72 h. After the incubation, aliquots of 2 mL of eggs were seeded on the diet in plastic trays (34 cm length × 24 cm width × 7 cm height), each containing 2 kg of CENA’s diet (Table S1), and the larvae were allowed to develop for 8 days at 25 °C. After that, the 3rd instar larvae were washed from the diet, collected by manual sieving, and allowed to pupate in vermiculite at room temperature (26 ± 2 °C, 70–80% RH, and 10 h of photophase). Three egg batches, each from a different generation (13th to 15th generation), were used with three diet trays (replicates) per treatment seeded within each batch. Hatch rates of each batch were taken from nine samples per treatment with 300 to 400 eggs distributed on pieces of moistened black filter paper held for 7 days in closed Petri dishes at 24 °C. During sampling of the egg aliquots, the percentage of 1st instar larvae present right after incubation was also estimated as follows: (number of 1st instar larvae/total number of eggs and larvae in sample) × 100. The total numbers of larvae and pupae obtained from the diets were recorded. The mean pupal weight was assessed by using 50 pupae from each treatment, 2 days before adult emergence, and the same biological material was used to assess the emergence of adults (%) [35] and sex ratio (number of females ÷ total number of males and females) in Petri dishes [12].

### 2.2. Assessment of Egg Production in Ovipositing Cages with Different Adult Densities

To assess the temporal variation of egg production from ovipositing cages (50 cm length × 30 cm width × 100 cm height) with different adult densities, the flies were kept at five different densities, i.e., 0.1, 0.2, 0.3, 0.4, and 0.5 flies/cm^2^, comprising a range between relaxed and overpopulated conditions. These densities were equal to ca. 2080, 4160, 6240, 8320, and 10,400 flies per cage, respectively. The number of pupae loaded in each cage was adjusted to the mean adult emergence of the three last generations of pupal cohorts (e.g., 88.9–95.2%) to attain the specified adult density, i.e., cages were loaded with 58 to 390 mL of pupae per cage depending on the treatment (1 mL of pupae ≈ 33 pupae) (Figure S1). One side of the cages consisted of a red voile cloth panel covered on the outside with a thin layer of transparent silicon rubber (<0.5 mm thick) protected by a wet sponge cloth placed all along the oviposition chamber door (made of aluminum angles and a transparent PVC cover to which the sponge was attached) to avoid dehydration of the oviposited eggs. In the middle of the cage, two half-cut pipes (7 cm diameter) served as a food station (ca. 615 g of a mix of sugar/wheat germ/yeast Bionis YE MF at 3:1:1) and water supply (a cotton wad moistened with sodium benzoate solution at 1% to prevent fungal growth for as long as possible). The adult density was calculated by dividing the number of flies by the total resting surface area of the cage (19,000 cm^2^ of cage + 1799 cm^2^ of the food and water feeders). It was not expressed by volume (cm^3^), because despite the fact that flies can move across the internal space of the cage, they do not fill all the free space all the time, and at night all flies land and rest on the internal surfaces of the cage. Pupae from three cohorts of different generations were used, and the cages were distributed in a randomized design with three replicates for each density. The ovipositing cages were maintained in a room with controlled environmental conditions during the entire experiment (24 ± 2 °C, 60–70% RH, and 12 h of photophase). Females were attracted to the red voile cloth panel by four 20 W bright white linear LED tube light bulbs positioned 1.5 m from the cages. The females introduced their ovipositor through the red panel, leaving the eggs trapped on the external surface of the red voile. Eggs were gently washed every 8 h to estimate daily and total egg production (mL), egg hatch (%), and period (days) of egg production per cage (from the first oviposition detected on the red panels until egg-laying stopped or when the volume collected became less than 0.1 mL of eggs/day). To assess egg hatch, 300 to 400 eggs were counted on a moist black cloth, placed over a wet sponge in Petri dishes, and incubated for 7 days at 24 °C. Then, the number of hatched eggs was estimated (egg hatch (%) = (number of hatched eggs ÷ total number of eggs) × 100).

### 2.3. Evaluation of Larval Density and Comparison of Diets

CENA’s diet (Table S1) had been used during the colonization of the *Vacaria* strain, but it was still unclear which would be the most suitable egg-seeding density for mass-production purposes. To solve this issue, seven different densities were tested: 0.5, 0.7, 1.0, 1.3, 1.5, 1.7, and 2 mL of eggs per 1 kg of CENA’s diet (*diet test* 1). The aliquots of eggs (1 mL of eggs ≈ 11,700 eggs) were obtained from four batches of the mother colony during different generations and seeded after 48 h in the bubbling thermal bath at 25 °C on plastic trays (47 cm length × 30 cm width × 4 cm height) containing 3 kg of CENA’s diet (Figure S2).

The ingredients used to prepare 1 kg of CENA’s diet were 60 g of the hydrolyzed brewer’s yeast Brewcell (Biorigin, Lençois Paulista, Brazil), 60 g of yellow corn flour Yoki (General Mills Alimentos Ltd., Cambará, Brazil), 60 g of sugar Caravelas (Usina Colombo S/A, Ariranha, Brazil), 6 g of an iota carrageenan (Agargel Ltd., João Pessoa, Brazil), 800 mL of tap water, 1 g of sodium benzoate, 8 mL of a Nipagin solution (10 g methyl 4-hydroxybenzoate/100 mL 96% ethanol), and 4 mL of hydrochloric acid to maintain initial pH at about 3.4. To prepare the diet, the solid ingredients (with the exception of carrageenan) were initially mixed in a blender with 400 mL of water. The carrageenan was dissolved in 400 mL of boiling water, added to the blender together with the antioxidant and antimicrobial agents (sodium benzoate, Nipagin solution, and hydrochloric acid), and stirred for 2 min until homogenized. The liquid diet was distributed on the plastic trays where it could solidify.

The trays containing CENA’s diet seeded with the different egg densities were maintained in an environmentally controlled room (24 ± 2 °C, 60% RH, and 10 h of photophase) during the entire larval period, after which the prepupae were washed and transferred to vermiculite. Before this, a random sample of 50 larvae were weighed to determine the mean larval weight of each treatment. Numbers of larvae and pupae obtained from the trays were manually counted, and pupae were maintained in a dark room at 24 ± 2 °C and 60% RH. Pupal weight (mg), pupal period (days), adult emergence (%), and sex ratio were assessed using the same methodology as described for the test of incubation time for eggs. The diameter of pupa (mm) was estimated by measuring 20 pupae from each treatment with a pachymeter (Vernier Caliper 100 mm Pocket Mini Gem., CHBC). To estimate flight ability, 50 pupae from each treatment were placed at the bottom of black Plexiglas tubes (9 × 10 cm high) whose walls were coated with talcum powder. After emergence, the flies that had flown out of the tubes, the flies that remained in the tubes, and unemerged pupae were counted. Percentages of larvae and pupae recovered were estimated as described by FAO/IAEA/USDA [35].

In addition to the development of a colony of the *Vacaria* strain at CENA, the strain was also reared on Embrapa Grape & Wine using a different larval diet that was mainly based on corn flour (Table S1). Embrapa’s diet had been used for many years and proved to be suitable for small-scale production of *Ceratitis capitata* and *A. fraterculus*, including the *Vacaria* strain (Kovaleski, personal communication). The ingredients to prepare 1 kg of Embrapa’s diet were 300 g of yellow corn flour Yoki (General Mills Brasil Alimentos Ltd. Cambará, Brazil), 50 g of brewer’s yeast Brewcell (Biorigin, Lençóis Paulista, Brazil), 30 g of sugar Caravelas (Usina Colombo S/A, Ariranha, Brazil), 1120 mL of tap water (boiled at temperatures ≥ 99 °C), 2 g of sodium benzoate, 2 mL of Nipagin solution (10 g methyl 4-hydroxybenzoate/100 mL 96% ethanol), and 6 g of citric acid. For its preparation, all the solid ingredients and antimicrobial agents were manually mixed in a plastic bowl, and then 1120 mL of boiling water (≥99 °C) was added to dissolve the solids. The diet was stirred manually with a wooden spoon until a smooth consistency was attained. Before transferring to trays (Figure S3), it should be left cooling until all residual steam comes out. The pH of Embrapa’s diet after cooling was between 4.0 and 4.5.

To determine which of the two larval diets (Table S1) would be more suitable for mass rearing the *Vacaria* strain, another bioassay was carried out (*diet test* 2), comparing production yields and the quality of flies. For this test, plastic trays (47 cm length × 30 cm width × 4 cm height) containing 3 kg of Embrapa’s diet were seeded with 6 mL of eggs per tray (which was considered the optimal density at Embrapa) (Kovaleski, personal communication), while other trays with the same dimensions containing 3 kg of CENA’s diet were seeded with a suitable density chosen from the previous bioassay (i.e., 4.5 mL of eggs/3 kg of diet). The eggs were not seeded directly on the surface of the diets, but distributed on pieces of filter paper placed over the diets (Figures S2 and S3). The used eggs were obtained from two different generations (20th and 21st). The trays were kept in the same room (24 ± 2 °C, 60% RH, and 12 h of photophase) and distributed in a randomized design with four replicates in total for each diet. After larval development, prepupae from both diets were washed and transferred to vermiculite. The total number and weight of 3rd instar larvae and pupae, diameter of pupae (mm), duration (days) of the larval and pupal development, larval and pupal recovery (%), emergence of adults (%), sex ratio (♀/♂ + ♀), and flight ability (%) were recorded for each diet as previously described. The productivity (number of pupae/kg of diet, number of pupae produced per USD 1.00 of diet, and amount of diet (kg) needed to produce 1 million pupae) and costs (USD) were also assessed for both diets (Table S1). To estimate the cost of 1 million flying adults, an adult emergence of 85% and flight ability of 90% were considered.

### 2.4. Mating Compatibility Tests

Eight field cages (3 m diameter × 2 m high) were used for mating compatibility tests. A potted citrus tree (*Citrus sinensis* L. cv. Bahia) was placed in the center of each field cage. The trees, ca. 2 m in height and 1 m in a canopy diameter without flowers or fruits, were lightly pruned before the test and had not been treated with any chemicals. In each cage, 26 sterile males and 26 sterile females of the laboratory strain and 26 males and 26 females of a wild population were released. Pupae were irradiated with 40 Gy of gamma rays 2 days before adult emergence to obtain males with 99% sterility and females fully sterile [12]. At the time of the tests, the colony had been maintained for 24 generations at CENA without any refreshment with wild flies. The wild flies for the tests were obtained from pupae collected from infested pineapple guava (*Feijoa sellowiana* Berg) from Vacaria, Rio Grande do Sul, and multiplied in papaya for 1 generation to provide sufficient flies for the tests. The released sterile flies were 9–10 days old and the wild flies were 15–17 days old [12,36]. Forty-eight hours before the test, flies were marked individually with a small dot of water-based paint on the dorsal surface of the thorax [35]. On the day of the test, the males were released first into the cages to give them the opportunity to disperse, dead flies were replaced, and 20 min later the females were released. Observations were made from 8:00 to 10:00 a.m. [36] by one person per cage, and throughout the observation period, the mating pairs were collected in 30 mL glass vials for later identification. Each cage was considered as a replicate, with four replicates performed in one day and the other four on the next day. Sterile flies from a single generation were used. The index of sexual isolation (ISI), male and female relative performance indices (MRPI and FRPI), and the relative isolation index (RII) were estimated [35].

### 2.5. Data Analysis

One-way analysis of variance (ANOVA) and Tukey’s honestly significant difference (HSD) multiple comparison test were used for the statistical analyses of the quality control data of the egg incubation tests, i.e., egg hatch (%), 1st instar larvae (%) in the sample before the end of incubation, numbers of larvae and pupae, pupal weight, adult emergence (%), and sex ratio.

To estimate the daily volume of eggs (mL) produced in cages with different adult densities during the entire oviposition period, the 3-parameter exponential function *y* = *a* + *bx* + *ce*^−*x*^ was fitted to the dataset of mean daily egg production per cage, where *a*, *b*, and *c* are nonzero parameters; daily egg production per cage (mL) is the response variable *y*; and time (day) is the predictor variable *x*, with a Gaussian distribution for the errors [37]. The mean daily and total egg production per cage and mean egg hatch for the period between day 1 and day 30 of oviposition were compared with Tukey’s test (α = 0.05).

After visual inspection of the data, quadratic regression model equations were obtained to describe the effect of the seven egg-seeding densities (*diet test* 1) on number of larvae and pupae, larval and pupal recovery (%), and egg–pupa recovery (%) (*y* = *a* + *bx* + *cx*^2^, where *a*, *b*, and *c* are constants; the response variable *y* is the quality control parameter; and *x* is the egg density), while linear regression equations were used for the datasets of larval and pupal weights (mg), larval and pupal periods (days), diameter of pupa (mm), adult emergence (%), sex ratio, and flight ability (%) (*y* = *a* + *bx*, where *a* is the *y*-intercept, *b* is the slope, *y* is the quality control parameter, and *x* is the egg density). The means of the 13 quality control parameters obtained from the larval diets of Embrapa and CENA (*diet test* 2) were compared separately by the Student’s *t*-test (α = 0.05). The one-sample *t*-test was used to verify if the mean values of the ISI, MRPI, and FRPI indices significantly differed from 0, or 1 in the case of the RII (α = 0.01).

The assumptions of homoscedasticity and normality were verified through the tests of Bartlett and Shapiro–Wilk, respectively [38,39]. The goodness of fit from the exponential, quadratic, and linear regression models was verified by the coefficient of determination (*r*^2^) [40]. The analyses were performed using the packages ‘Agricolae’, ‘nlraa’, ‘easyreg’, and ‘MLmetrics’ in the statistical environment R [41].

## 3. Results

### 3.1. Incubation Time of the Eggs in Water

The results of the experiment of the different incubation times of the eggs in the bubbling thermal bath are summarized in Table 1. Significant differences were observed for viability of eggs, presence of first instar larvae immediately after incubation, and number of pupae produced (*p* < 0.05). After 7 days in Petri dishes, egg hatch was higher at the incubation time of 48 h as compared with the other incubation times (F_2,24_ = 10.1; *p* = 0.01). On the day of egg sampling, the percentages of first instar larvae present in the egg aliquots sampled from the treatments of 60 and 72 h were around 50%, indicating that hatching had already started in these two treatments. The high hatch rate before the end of incubation reduced larval and pupal yields, as fewer larvae and pupae were recovered in the 72 h treatment (Table 1). Considering the number of larvae, there was no significant difference among treatments (F_2,24_ = 3.6; *p* = 0.09). Nevertheless, more third instar larvae were obtained after 48 h of bubbling, and there was a high percentage of undesired second instar larvae in trays that had been seeded with eggs from the 72 h treatment (ca. 5–10% of the larval cohort).

### 3.2. Egg Production in Oviposition Cages with Different Adult Densities

**Figure 1:**
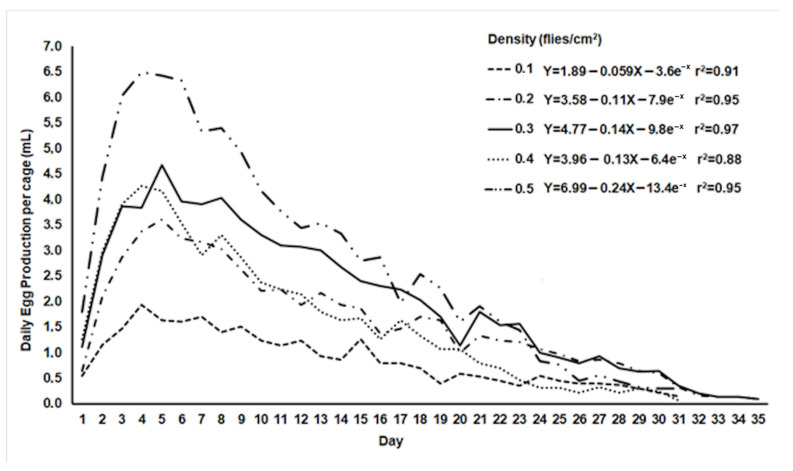
Observed mean volume of eggs collected daily from cages with *Anastrepha fraterculus* flies at five different adult densities.

**Figure 2:**
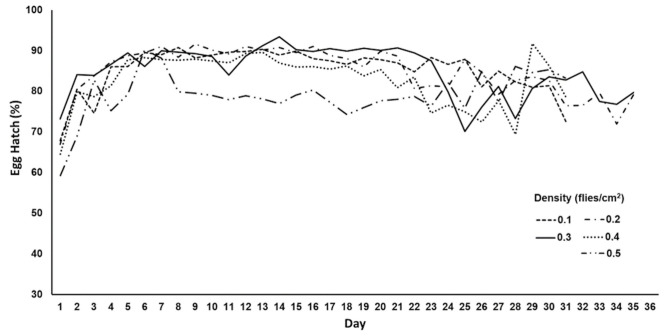
Mean daily hatch of eggs produced by *Anastrepha fraterculus* flies maintained at five different adult densities.

Both egg production and egg hatch were affected by increasing adult fly densities in oviposition cages (Figure 1 and Figure 2). The volume of eggs collected daily per cage increased as the number of flies/cm^2^ increased (Figure 1). According to the exponential equations obtained, the production peak would occur on the 4th day after the females started laying eggs at the increasing densities (predicted values of 1.61, 3.1, 4.1, 3.4, and 5.9 mL of eggs/cage/day), but the peaks for the 0.2 and 0.3 densities were observed on the 5th day (3.6 and 4.7 mL of eggs/cage/day, respectively) (Figure 1). After the 4th or 5th day of oviposition, the daily egg production declined in all treatments. Females from all treatments continued laying eggs up to 31 days, but females from the 0.2 and 0.3 density treatments produced more than 0.1 mL of eggs/cage/day up to 35 days. These two densities produced ca. 0.6 mL of eggs/cage/day on day 30, while the cages from the other densities were providing 0.3 mL of eggs/day or less (Figure 1).

Considering the mean daily egg production per cage between day 1 and day 30 of oviposition, the mean production of the 0.5 density treatment (2.94 ± 0.4 mL of eggs/cage/day) differed statistically from the 0.1 density treatment (0.9 ± 0.04 mL of eggs) but not from the 0.3 density treatment (2.31 ± 0.2 mL of eggs) (Table 2). There were no significant differences between the mean production of the 0.2 and 0.4 density treatments (Table 2), and their curves overlapped on most of the days after the 6th day of oviposition (Figure 1). Despite the high value of total egg production observed at the 0.5 density (88.1 ± 6.9 mL of eggs), it differed significantly only from the 0.1 treatment (26.01 ± 7.1 mL of eggs) (Table 2).

During the 30 days of oviposition, several mean egg hatch values overlapped (Figure 2), the mean daily values ranged between 78% and 88%, and all means remained above 65% until day 30. The mean egg hatch rate observed at the 0.5 density treatment (78.9 ± 0.7%) was slightly lower than the means detected at the 0.1, 0.2, and 0.3 density treatments, but it did not differ from that recorded at the 0.4 density treatment (82.8 ± 1.2%) (Table 2).

### 3.3. Larval Diets

**Figure 3:**
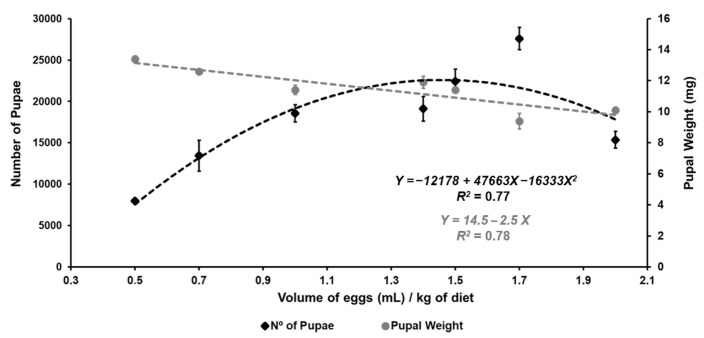
Regression lines for the number of pupae and pupal weight obtained from a diet with corn flour and carrageenan (CENA’s diet) at different egg-seeding densities. Error bars represent the standard errors of means.

Data on the quality control parameters of flies reared on CENA’s diet (*diet test* 1) are shown in Table 3 and Figure 3. The seven different egg-seeding densities significantly affected most of the parameters, except the diameter of pupae, pupal period, adult emergence, sex ratio, and flight ability (*p* > 0.05). (Table 3). The best yields were obtained using densities of 1.0 and 1.7 mL of eggs/kg of diet. Based on the quadratic regressions, the maximum number of pupae was obtained with the estimated density of 1.46 mL of eggs/kg, while the highest pupal and egg–pupa recoveries were obtained with densities of 0.98 and 0.9 mL of eggs/kg, respectively. The lowest mean egg–pupa recovery was found with the 2.0 density (<40%). As the density increased, the number of pupae increased up to the 1.7 density, but the pupal weight decreased (Figure 3). Pupae weighing ≥ 11 mg could only be obtained with a density of up to 1.5 mL of eggs/kg of diet. Based on the overall results, the densities of 1.0 and 1.5 mL of eggs/kg of diet were adequate to produce high numbers of good quality flies, while the 2 mL density proved to be unsuitable for rearing the *Vacaria* strain in this larval diet.

In *diet test* 2, the larval diet that has been used to maintain a small colony of the *Vacaria* strain at Embrapa was compared with CENA’s diet (Table 4). The two diets differed significantly with respect to the numbers of larvae and pupae produced, larval and pupal recovery, pupal weight and diameter, egg–pupa recovery, and adult emergence (*p* < 0.05). More larvae and pupae were obtained with CENA’s diet (ca. 3 to 4 times more), but Embrapa’s diet produced bigger and heavier pupae (Table 4). The parameters of larval weight, larval and pupal periods, sex ratio, and flight ability were not affected by the type of diet. Comparing the unit cost of the two diets, CENA’s diet was cheaper, allowing almost a 4-fold increase in pupal production per kg of diet (Table 5). Production of 1 million pupae would require 133.6 kg of CENA’s diet (at a cost of USD 58.78) but 457.7 kg of Embrapa’s diet (USD 210.54) (Table 5).

### 3.4. Mating Compatibility

**Figure 4:**
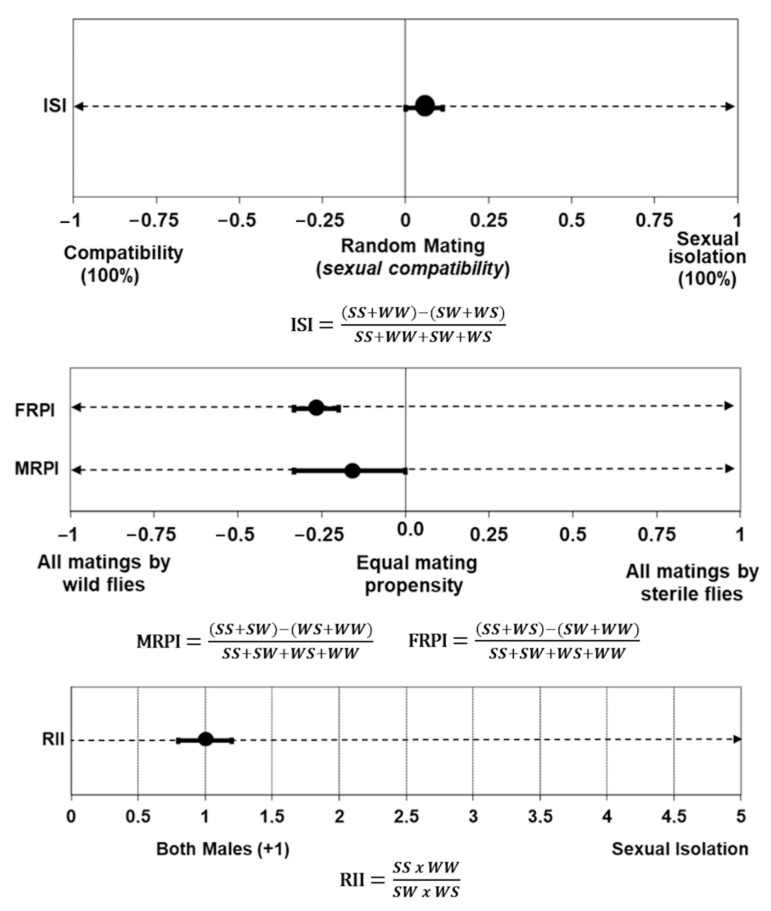
Indices of mating compatibility between sterile flies of the *Vacaria* strain and wild flies of *Anastrepha fraterculus* (ISI = isolation index; FRPI = female relative performance index; MRPI = male relative performance index; RII = relative isolation index; *SS* = sterile male and female; *WW* = wild male and female; *SW* = sterile male and wild female; *WS* = wild male and sterile female). Error bars represent the standard errors of means.

Out of 416 possible pairs in 8 field cages, 225 copulations were obtained. The index of sexual isolation (ISI) of 0.055 ± 0.046 suggested that wild females mated randomly with wild or sterile males (*t* = 1.7; *p* = 0.24), indicating an acceptable level of sexual compatibility between both types of flies (Figure 4). The mean relative isolation index (RII) of 1.0 ± 0.25 also indicated random mating (*t* = 0.37; *p* = 0.72).

Sterile males from the Vacaria strain were as effective at obtaining mates as wild males (Figure 4), having a mean MRPI of −0.17 ± 0.16 (*t* = 1.7; *p* = 0.23). The mean FRPI (−0.27 ± 0.06) was also close to zero (*t* = 8.7; *p* = 0.013), indicating that sterile females were slightly less competitive than wild females. Therefore, no evidence of sexual isolation was found between wild females from the target population and the mass-reared sterile males.

## 4. Discussion

Improving the mass-rearing techniques of a target insect pest species is a critical step for the implementation of the SIT, as it may allow high production of good quality insects at the lowest possible cost [28]. In the genus *Anastrepha*, some species have been successfully mass-reared, such as *A. suspensa* and *Anastrepha ludens* in the United States [42,43]; *A. ludens*, *Anastrepha obliqua*, and *Anastrepha serpentina* in Mexico [44,45,46]; and *A. fraterculus* in Argentina and Brazil [15,17]. However, the mass-rearing system developed in Brazil for *A. fraterculus* was not optimal as the reported mean egg–pupa recovery was only 17% [17]. Additionally, after 60 generations without any introduction of wild flies, the Brazilian strain (i.e., the *Piracicaba* strain) showed evidence of reduced mating compatibility with other wild strains and populations [27]. To address these problems as well as the need of the Moscasul project for a large number of sterile and high-quality flies [13], this study aimed to refine some key components of mass-rearing protocols for a recently established *A. fraterculus* strain originating from the target southern population.

To diminish dehydration and guarantee a more uniform egg hatch, *Anastrepha* eggs must be incubated in water for a certain period [14]. In Mexico, the eggs from *A. ludens* are incubated for 90 h in a bubbling bath at 25–26 °C, while the *A. obliqua* eggs are incubated for 61 h at 27 °C [45]. In Argentina and Brazil, *A. fraterculus* have usually been air-bubbled in water for 48 h [16,17]. Our results showed that incubation times greater than 48 h in water at 25 °C can reduce egg hatch or increase larval mortality, resulting in lower pupal yields (Table 1). Therefore, 48 h of incubation in a bubbling system at 25 °C is the recommended protocol for the *Vacaria* strain before seeding.

In mass-rearing facilities, adult fruit flies are often confined in cages of diverse designs depending on the number of flies to be produced and cage function, e.g., mother colony under relaxed conditions or release colony [28,47,48]. Mother colonies under relaxed rearing conditions have been used in some facilities to maintain the vigor and size of strains of *C. capitata*, *A. obliqua,* and *A. ludens* [47,48,49,50], thus preventing the rapid accumulation of deleterious traits. Based on our findings, the adult density of 0.3 flies/cm^2^ could be recommended for the mother colony cages of the *Vacaria* strain (Figure 1 and Figure 2). Flies kept in those cages at this density produced more eggs per day than cages with 0.4 flies/cm^2^ (Table 2). In addition, its mean daily egg production (2.31 ± 0.2 mL of eggs/cage/day) did not differ significantly from the production of cages with 0.5 flies/cm^2^ (2.94 ± 0.4 mL of eggs/cage/day) during the 30 days of oviposition. The mean daily volumes of eggs collected per adult colony cage reported by Vera et al. [16] and Walder et al. [17] were 1.9–4.3 mL (cage 60 cm length × 30 cm width × 96 cm height with 0.48 flies/cm^2^) and 0.42–5.6 mL of eggs (cage 50 cm length × 30 cm width × 100 cm height with 0.36 flies/cm^2^), respectively, with egg hatch values around 84% and 64%. Orozco-Davila et al. [50] also reported an increased daily fecundity for *A. obliqua* in cages with a lower density of ca. 0.6 flies/cm^2^. Despite a slightly lower mean hatchability of eggs (Table 2), a density of 0.5 flies/cm^2^ could be applied in mass-rearing egging cages to obtain a greater amount of eggs (>5 mL/cage) in a shorter period of time (<7 days) to enhance the production of sterile flies for field releases, but not for the production of flies for the maintenance of the colony.

Another very important and costly component of the rearing is the larval diet. By replacing agar with carrageenan and wheat germ with corn flour, CENA’s diet became cheaper than the diet proposed by Walder et al. [17] (USD 0.44 and USD 0.52/kg of diet, respectively) (Table S1) but still suitable for the production of large numbers of good quality flies at egg-seeding densities between 1 and 1.7 mL of eggs/kg of diet. Up to a density of 1.7 mL of eggs/kg, the mean egg–pupa recovery with CENA’s diet ranged from 57 to 74% (Table 3), which is higher than the mean recovery values of 56% reported by Vera et al. [16] and 17% reported by Walder et al. [17]. Vera et al. [51] obtained an egg–pupa recovery close to 45% in a liquid diet with sponge cloth support. Using the 1.5 mL of eggs/kg density, the number of pupae produced per kg of larval diet (7484 pupae/kg) (Table 4 and Table 5) was also higher than the values reported by Gonzalez et al. [52], Vera et al. [16], and Walder et al. [17] (i.e., 876, 6843, and 2630 pupae/kg of diet, respectively), but Vera et al. [51] obtained up to 11,720 pupae/kg in a liquid diet. The percentages of adult emergence and flyers were higher than 90% and 70% (Table 3 and Table 4), which are above or similar to the minima pre-irradiation values accepted by the FAO/IAEA/USDA [35] for *A. ludens* (80% and 88%), *A. obliqua* (92% and 89%), and *A. suspensa* (85% and 75%). Walder et al. [17] reported a mean emergence of 85% and flight ability ranging from 46 to 74% for the *Piracicaba* strain of *A. fraterculus*.

The weight of pupae produced with CENA’s diet at densities between 1 and 1.5 mL of eggs/kg of diet was around 11.4 mg, which is less than the mean values reported for *A. fraterculus* by Gonzalez et al. [52] (13 mg), Vera et al. [16,51] (13.1 and 13.7 mg), and Walder et al. [17] (14.3 mg). The Embrapa diet also produced heavier pupae (15.6 ± 1.1 mg); however, as a result of the low larval recovery (~11.5%) (Table 4), the larval density was low during development, which in turn reduced competition stress in the surviving larvae (Figure S3). Further investigation is needed to verify that a weight of about 11 mg is acceptable for an SIT program against *A. fraterculus*, because male size can influence mating success in some *Anastrepha* species such as *A. suspensa* and *A. obliqua* [53,54,55,56].

It is worth mentioning that the nutritional balance and quality of the larval diets from CENA and Embrapa were not the same (Table S2). The initial carbohydrate/protein ratio was 2-fold higher in Embrapa’s diet (ca. 6:1). Furthermore, the approximated amounts (*w*/*w*) of proteins, carbohydrates, lipids, and fibers provided by the main raw ingredients were 1.4, 2.5, 4.4, and 3.2 times higher in Embrapa’s diet, respectively (Table S2). The excess or imbalance of some nutrients can be detrimental for the larval development of tephritids [57,58]. Fruit fly larvae are efficient users of available proteins even at concentrations as low as 2%, and concentrations higher than 8% can exceed optimal quantities needed [51,59,60,61]. Regarding carbohydrates, Vera et al. [51] observed that *A. fraterculus* larvae were unable to complete their development in liquid diets with more than 40% sugar. In contrast, Pascacio-Villafan et al. [62] reported that *A. ludens* reared on carbohydrate-biased diets exhibited longer larval and pupal periods, but heavier pupae. High concentrations of crude lipids and vitamins can also inhibit the larval development of *A. obliqua* [59].

Theoretically, the nutrients from the raw ingredients used in Embrapa’s diet should have been sufficient to produce large quantities of good quality flies, but *diet test* 2 demonstrated that larval recovery remained very low (<15%) (Table 4). The soft texture of the diet and low pH were probably not a problem, because feeding larvae tunneled freely in the diet, and their guts can vary from acidic to neutral in nature (pH 3–7) [57,63,64]. The higher contents of crude proteins, carbohydrates, and lipids (Table S2), or even other factors related to changes in nutrient interactions or bioavailability provoked by cooking with boiling water [46,57], might have affected larval development more. The rearing scale is also another important factor to consider as Embrapa’s diet had been previously used only in small containers, usually with 500–700 g of diet, at Vacaria (Barros, personal communication). Some studies have reported possible dietary modifications caused by scaling from laboratory to mass-rearing levels [65,66]. For example, Moreno et al. [59] verified that diets containing carrot, Glymaxene, and corn germ cake were suitable for rearing *A. obliqua* larvae in cups with 100 g of diet, but the results were not satisfactory when trays with 750 g of diet were used.

As the actual content of proteins, lipids, carbohydrates, vitamins, and minerals in the tested diets or their bioavailability after diet preparation were not determined, we are reticent to render definitive conclusions about the exact effects of dietary components and other factors on the quality control parameters evaluated. Rather, the productivity results from *diet test* 2 allowed us to decide which diet would be best for the mass rearing of the *Vacaria* strain. In view of the low egg–pupa recovery and its high cost (Table 4 and Table 5), Embrapa’s diet in its current formulation could be recommended only for small-scale laboratory rearing of the *Vacaria* strain, although it is simple to prepare.

Further studies could improve the performance of the *Vacaria* strain on both diets and reduce their costs. For example, potential benefits from varying the levels of brewer’s yeast or corn flour in the diets should be investigated. Carrageenan is cheaper than agar but at USD 20/kg it is still expensive in Brazil and requires high temperature to be activated, which encourages additional research to replace it with other bulking agents, such as guar gum, or the development of novel diets with cheaper local ingredients.

Despite the relatively high cost of some of the ingredients of CENA’s diet, its unit cost of ca. USD 80 to produce 1 million *A. fraterculus* flies is not unrealistic for an SIT program as, for example, the total cost for the same amount of sterile *C. capitata* is ca. USD 250 (including the costs of adult and larval diets, labor, operational costs, etc.) [11]. Pascacio-Villafan et al. [67] estimated that the cost to produce 1 million *A. ludens* flies with yeast-reduced agar and carrageenan gel diets would be around USD 200. 

Even after two years under laboratory rearing conditions, and without any introduction of wild flies, the sterile males from the *Vacaria* strain were proven to be compatible with wild females from Vacaria, and no evidence of sexual isolation was found between the sterile males and wild flies (Figure 4). Wild females mated more than the sterile females, which was expected since females are more radiosensitive. However, this is of no great concern as sterile females play no role in the induction of sterility into the wild population [12,28]. Perhaps the avoidance of overpopulated conditions as recommended by the new rearing protocols might have contributed to a low accumulation of unfavorable sexual traits or prevented them over the 24 generations, but there could be other factors acting concurrently, such as the genetic background of the strain or low genetic drift [28,68]. Additional field cage tests with wild and sterile flies from future generations of the *Vacaria* strain should be carried out to continue monitoring the mating behavior of the sterile males over time.

As the facility of the Moscasul project currently does not have an irradiator, CENA has built up its rearing to supply sterile flies for dispersion trials and pilot projects for wild population suppression in the Sierra Gaucha [13]. Between the 1st and 24th generations, CENA produced 174.9 L of pupae (≈5.8 million flies) of the *Vacaria* strain, with a mean of 7.3 L of pupae per generation. In the 2019–2020 season, ca. 7 to 10 L of sterile pupae were sent every month by air to Vacaria for the release of sterile flies in forest areas surrounding commercial apple orchards. Field trials are currently being carried out to evaluate the efficacy of using sterile flies from the *Vacaria* strain to suppress wild *A. fraterculus* populations in southern Brazil.

## 5. Conclusions

Based on the results of this study, it is recommended to incubate *A. fraterculus* eggs for 48 h in a bubbling bath at 25 °C, and an adult density of 0.3 flies/cm^2^ can be used in mother colony cages, while 0.5 flies/cm^2^ could be used in mass-rearing egging cages to obtain large volumes of eggs in a shorter period of time. Large numbers of good quality flies can be obtained with a larval diet containing corn flour and carrageenan (CENA’s diet) at egg-seeding densities between 1 and 1.5 mL of eggs/kg of diet. Two years of fly production following the rearing protocols described here did not induce mating incompatibility between sterile flies of the *Vacaria* strain and wild flies from the target population.

## Figures and Tables

**Table 1:** Quality control parameters (mean ± SE) obtained after the incubation of *Anastrepha fraterculus* eggs at three different time periods in a bubbling thermal bath system at 25 °C.

Quality Control Parameters	No. of Hours under Aeration			ANOVA
	48 h	60 h	72 h	
Egg hatch (%)	83.4 ± 3.6 a ^1^	49.8 ± 8.0 b	55.4 ± 4.6 b	*F*_2,24_ = 10.1; *p* = 0.01
First instar larvae before the end of incubation (%)	0.9 ± 0.8 a	50.8 ± 4.6 b	50.7 ± 3.6 b	*F*_2,24_ = 71.5; *p* < 10^−3^
Number of larvae	10,880 ± 1251 a ^2^	9560 ± 941 a	7360 ± 424 a	*F*_2,24_ = 3.6; *p* = 0.09
Number of pupae	7100 ± 662 a	6200 ± 391 ab	4450 ± 181 b	*F*_2,24_ = 8.8; *p* = 0.02
Pupal weight (mg)	10.4 ± 0.3 a	9.8 ± 0.2 a	10.3 ± 0.1 a	*F*_2,24_ = 2.3; *p* = 0.19
Adult emergence (%)	89.0 ± 5.0 a	82.7 ± 1.9 a	90.7 ± 2.4 a	*F*_2,24_ = 1.6; *p* = 0.29
Sex ratio (♀/♂ + ♀)	0.5 ± 0.02 a	0.5 ± 0.01 a	0.6 ± 0.03 a	*F*_2,24_ = 4.9; *p* = 0.053

^1^ Means (± SE) followed by the same letters in the lines do not differ significantly by Tukey’s test (*p* > 0.05). ^2^ Original means in this table. The numbers of larvae and pupae were transformed by log(x + k) before the analyses.

**Table 2:** Mean daily and total egg production per cage and egg hatch (mean ± SE) for the period between day 1 and day 30 of oviposition from cages with *Anastrepha fraterculus* flies at different adult densities.

Adult Density (Flies/cm^2^)	Daily Egg Production (mL)	Total Egg Production (mL)	Egg Hatch (%)
0.1	0.9 ± 0.042 c ^1^	26.01 ± 7.1 a	85.8 ± 0.8 a
0.2	1.79 ± 0.17 bc	53.5 ± 13.9 ab	86.2 ± 0.9 a
0.3	2.31 ± 0.2 ab	69.1 ± 14.2 ab	85.7 ± 1.1 a
0.4	1.71 ± 0.2 bc	50.5 ± 14.2 ab	82.8 ± 1.2 ab
0.5	2.94 ± 0.4 a	88.1 ± 6.9 b	78.9 ± 0.7 b
**ANOVA**	F_4,145_ = 10.3; *p* < 10^−3^	F_4,40_ = 3.8; *p* = 0.038	F_4,145_ = 8.6; *p* < 10^−3^

^1^ Means (± SE) followed by the same letters in the columns do not differ significantly by Tukey’s test (*p* > 0.05).

**Table 3:** Quality control parameters (mean ± SE) for *Anastrepha fraterculus* reared on a larval diet with corn flour and carrageenan (CENA’s diet) at seven different egg-seeding densities.

Quality Control Parameters	Density (mL of Eggs/kg of Diet)							Regression Analyses
	0.5	0.7	1.0	1.3	1.5	1.7	2.0	
Number of larvae	8761 ± 373	12,013 ± 1326	22,312 ± 2033	19,271 ± 1658	28,147 ± 1303	27,668 ± 1358	23,442± 1385	Y = −5029.4 + 29,499.3 X − 6778.8 X^2^, r^2^ = 0.91
Larval weight (mg)	19.4 ± 0.3	19.7 ± 0.4	17.6 ± 0.1	17.6 ± 0.6	17.4 ± 0.5	16.8 ± 0.9	14.2 ± 0.5	Y = 21.4 − 3.1 X, r^2^ = 0.86
Number of pupae	7959 ± 244	13,448 ± 1859	18,557 ± 1016	19,100 ± 1493	22,451 ± 1439	27,593 ± 1364	15,357± 1022	Y = −12,178 + 47,663 X −16,333 X^2^, r^2^ = 0.77
Pupal weight (mg)	13.4 ±0.2	12.6 ±0.1	11.4 ±0.3	11.9 ± 0.4	11.4 ±0.2	9.4 ± 0.5	10.09 ± 0.2	Y =14.5 − 2.5 X, r^2^ = 0.78
Larval recovery (%)	67.7 ± 2.9	79.0 ± 11.1	86.1 ± 7.9	58.7 ± 5.1	72.5 ± 3.4	62.8 ± 3.1	45.3 ± 2.7	Y = 45.4 + 68.9 X − 34.8 X^2^, r^2^ = 0.84
Larval period (days)	8.0 ± 0.0	8.0 ± 0.0	7.7 ± 0.3	7.0 ± 0.0	7.3 ± 0.3	7.0 ± 0.0	7.0 ± 0.0	Y = 8.4 − 0.79 X, r^2^ = 0.88
Diameter of pupa (mm)	2.2 ± 0.01	2.2 ± 0.03	2.1 ± 0.02	2.2 ± 0.03	2.1 ± 0.04	2.0 ± 0.03	2.1 ± 0.03	Linear regression not significant (*p* = 0.11)
Pupal recovery (%)	91.0 ± 1.6	94.0 ± 0.6	84.3 ± 4.3	99.3 ± 0.73	79.6 ± 2.0	99.7 ± 0.3	65.4 ± 0.6	Y = 71.2 + 48.6 X − 24.9 X^2^, r^2^ = 0.63
Egg–pupa recovery (%)	61.5 ± 1.9	74.2 ± 10.3	71.7 ± 3.9	58.2 ± 4.6	57.8 ± 3.7	62.7 ± 3.1	29.7 ± 2.0	Y = 39.7 + 67.5 X − 35.8 X^2^, r^2^ = 0.89
Pupal period (days)	15.0 ± 0.0	15.0 ± 0.0	14.0 ± 0.0	14.3 ± 0.3	15.3 ± 0.3	14.3 ± 0.3	15.0 ± 0.0	Linear regression not significant (*p* = 0.98)
Adult emergence (%)	92.3 ± 1.0	96.7 ± 2.0	91.7 ± 1.4	95.1 ± 1.9	94.7 ± 0.7	90.7 ± 1.9	93.0 ± 1.3	Linear regression not significant (*p* = 0.64)
Sex ratio (♀/♂ + ♀)	0.50 ± 0.02	0.55 ± 0.02	0.50 ± 0.03	0.57 ± 0.04	0.52 ± 0.03	.53 ± 0.05	0.55 ± 0.03	Linear regression not significant (*p* = 0.34)
Flight ability (%)	72.1 ± 0.2	75.0 ± 1.6	70.9 ± 1.1	74.3 ± 1.6	73.3 ± 0.6	71.2 ± 1.2	73.0 ± 0.8	Linear regression not significant (*p* = 0.85)

**Table 4:** Quality control parameters (mean ± SE) for *Anastrepha fraterculus* reared on a larval diet based on corn flour (Embrapa’s diet) or corn flour and carrageenan (CENA’s diet).

Quality Control Parameters	Larval Diets		ANOVA
	Embrapa	CENA	
No. of larvae	6688 ± 297 b ^1,2^	28,147 ± 1303 a	F_1,5_ = 1280.3; *p* < 10^−4^
Larval weight (mg)	17.8 ± 0.2 a	17.4 ± 0.5 a	F_1,6_ = 1.1; *p* = 0.33
Larval recovery (%)	11.5 ± 0.5 b	72.5 ± 3.4 a	F_1,5_ = 1732.5; *p* < 10^−4^
Larval period (days)	8.0 ± 0.0 a	7.3 ± 0.3 a	F_1,6_ = 6.43; *p* = 0.0522
No. of pupae	6554 ± 134 b	22,451 ± 1439 a	F_1,6_ = 181.7; *p* < 10^−4^
Pupal weight (mg)	15.6 ± 1.1 a	11.4 ± 0.2 b	F_1,5_ = 71.03; *p* = 0.0011
Diameter of pupa (mm)	2.3 ± 0.04 a	2.1 ± 0.04 b	F_1,6_ = 8.03; *p* = 0.0365
Pupal recovery (%)	98.0 ± 1.0 a	79.6 ± 2.0 b	F_1,6_ = 75.76; *p* = 0.0003
Egg–pupa recovery (%)	11.5 ± 0.5 b	57.8 ± 3.7 a	F_1,5_ = 4634.22; *p* < 10^−4^
Pupal period (days)	16.0 ± 0.0 a	15.3 ± 0.3 a	F_1,6_ = 6.43; *p* = 0.0522
Emergence (%)	88.7 ± 1.7 b	94.7 ± 0.7 a	F_1,6_ = 13.86; *p* = 0.0137
Sex ratio (♀/♂ + ♀)	0.46 ± 0.03 a	0.52 ± 0.03 a	F_1,6_ = 1.39; *p* = 0.2920
Flight ability (%)	69.1 ± 14.8 a	74.0 ± 0.8 a	F_1,5_ = 1.81; *p* = 0.1697

^1^ Means (± SE) followed by the same letters in the lines do not differ significantly by the Student’s *t*-test (*p* > 0.05). ^2^ Original means in this table. The numbers of larvae and pupae were transformed by log(*x* + k) before the analyses.

**Table 5:** Data of productivity and cost of the two larval diets tested for the *Vacaria* strain of *Anastrepha fraterculus*.

Larval Diet	USD/kg of Diet ^1^	Pupae/kg of Diet ^2^	Pupae/USD 1.00 of Diet ^3^	kg of Diet for 1 Million Pupae ^4^	USD per 1 Million Pupae ^5^	USD per 1 Million Flying Adults ^6^
Embrapa	0.46	2185	4750.0	457.7	210.54	275.22
CENA	0.44	7484	17,009.1	133.6	58.78	76.84

^1^ The cost per kg of diet was obtained by summing the costs of each ingredient used (Table S1). ^2^ To estimate the number of pupae per 1 kg of diet, the mean number of pupae from each diet (Table 4) was divided by 3. ^3^ Pupae per USD 1.00 of diet = (1 ÷ USD/kg of diet) × (pupae/kg of diet). ^4^ kg of diet for 1 million pupae = 10^6^ ÷ (pupae/kg of diet). ^5^ USD per 1 million pupae = (USD/kg of diet) × (kg of diet for 1 million pupae). ^6^ USD per 1 million flying adults = (10^6^ × USD per 1 million pupae) ÷ (10^6^ × 0.85 × 0.90).

## Data Availability

Data available within the article or its Supplementary Materials.

## Supplementary Materials

The following are available online at https://www.mdpi.com/article/10.3390/insects12070622/s1, Figure S1: Cages used for the assessment of egg production with different adult densities. The useful area and volume for flies were 20,799.00 cm^2^ and 148,076.75 cm^3^, respectively; Figure S2: Larval diet with corn flour and carrageenan (CENA’s diet) seeded with *Anastrepha fraterculus* eggs (at the left) and third instar larvae (at the right); Figure S3: Larval diet based on corn flour (Embrapa’s diet) seeded with *Anastrepha fraterculus* eggs (at the left) and appearance of the diet after larval development (at the right); Table S1: Amount of ingredients and their costs to prepare 1 kg of a larval diet based on corn flour (Embrapa’s diet) or corn flour and carrageenan (CENA’s diet); Table S2: Nutritional content of the main raw ingredients (according to suppliers’ information) and their approximate contribution to larval diets from Embrapa and CENA.
