# Supplementary material for: Improvement of the Mass-Rearing Protocols for the South American Fruit Fly for Application of the Sterile Insect Technique
Source: Insects. 2021 Jul 9;12(7):622. doi: 10.3390/insects12070622 (PMC8304162; doi:10.3390/insects12070622)
Supplement: Supplementary file 1 [file insects-12-00622-s001.zip › insects-1142990-fsup.pdf]

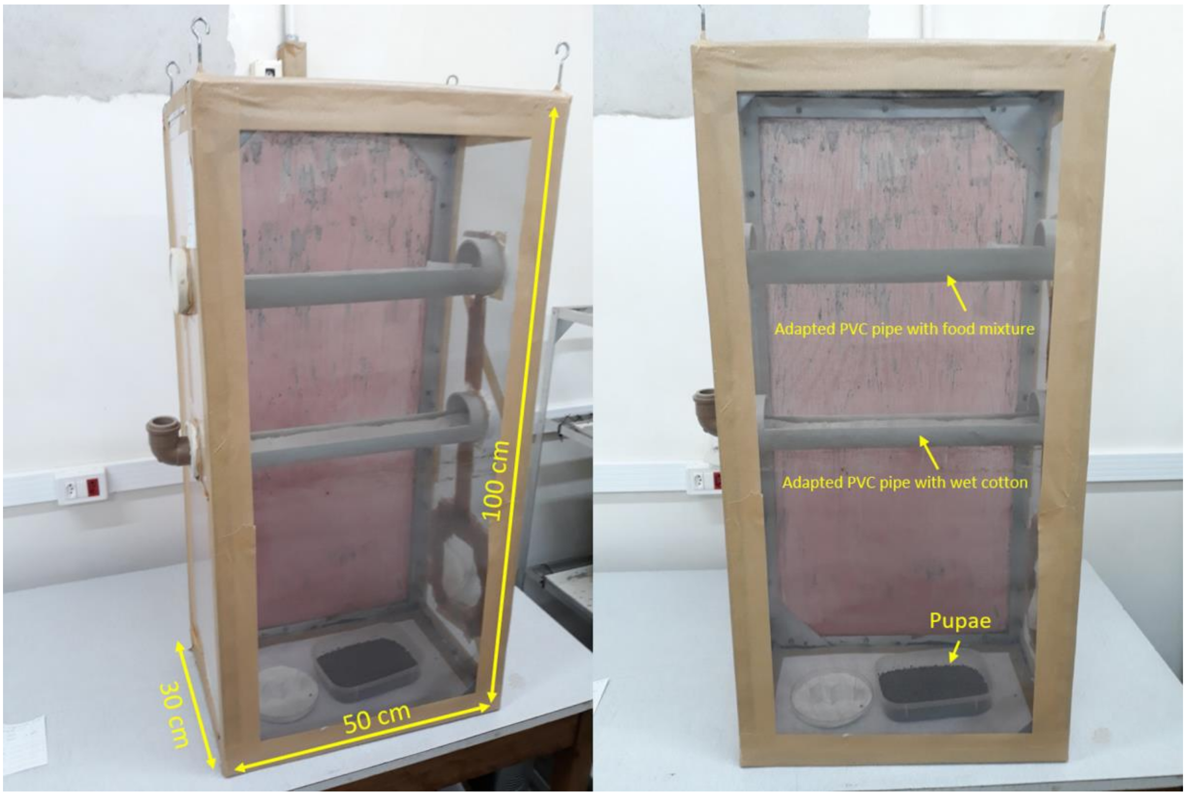

Figure S1. Cages used for the assessment of egg production with different adult densities. The useful area and volume for flies were 20,799.00 cm^2 and 148,076.75 cm^3, respectively.

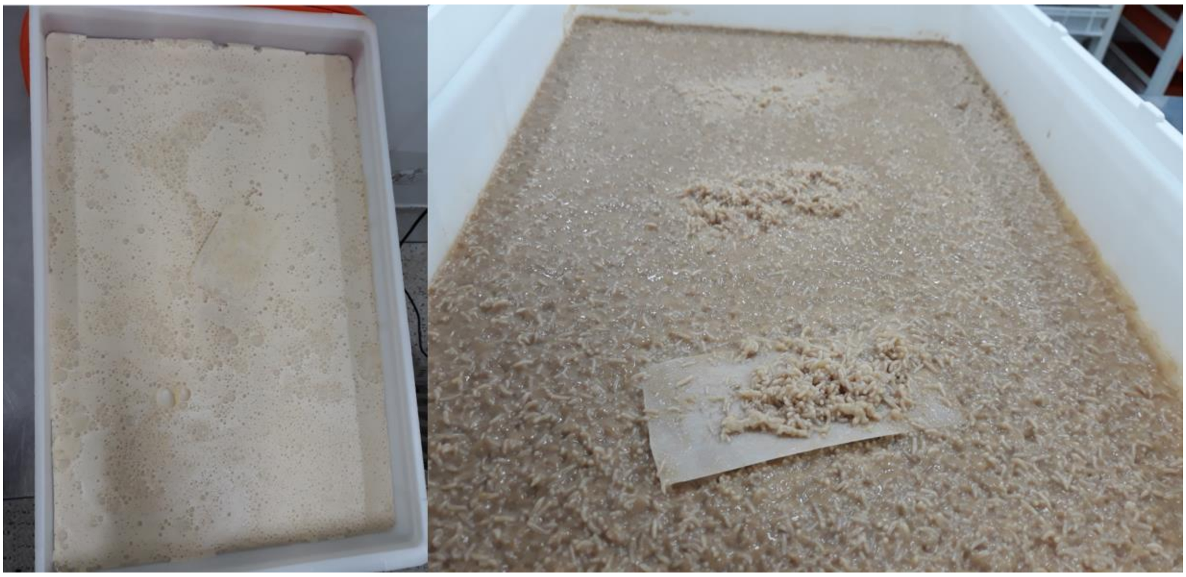

Figure S2. Larval diet with corn flour and carrageenan (CENA's diet) seeded with Anastrepha fraterculus eggs (at the left) and third instar larvae (at the right).

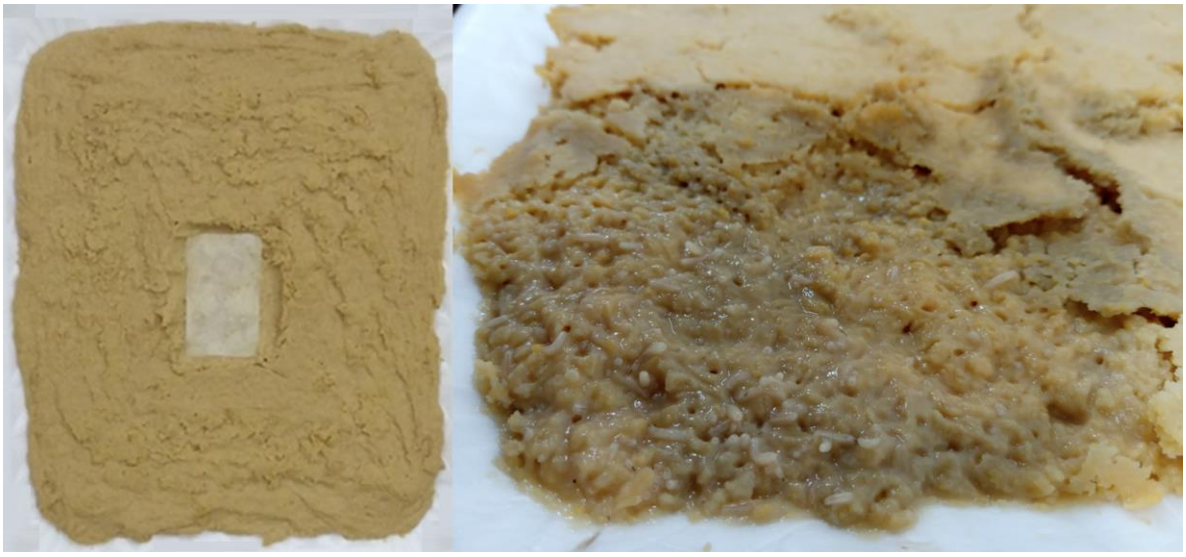

Figure S3. Larval diet based on corn flour (Embrapa's diet) seeded with Anastrepha fraterculus eggs (at the left) and appearance of the diet after larval development (at the right).

Table S1. Amount of ingredients and their costs to prepare 1 kg of a larval diet based on corn flour (Embrapa's diet) or corn flour and carrageenan (CENA's diet).

INGREDIENT	Embrapa's diet		CENA's diet	
	**Quantity (for 1 kg of diet)**	**Cost (US$) [1]**	**Quantity (for 1 kg of diet)**	**Cost (US$)**
Yellow corn flour Yoki®	300 g	0.28	60 g	0.053
Carrageenan Agargel®	---	---	6 g	0.17
Brewer's yeast Brewcell®	50 g	0.068	60 g	0.080
Sugar	30 g	0.043	60 g	0.089
Sodium benzoate	2 g	0.025	1 g	0.011
Nipagin solution 0.1%	2 mL	0.0028	8 mL	0.015
Citric acid	6 g	0.034	---	---
Hydrochloric acid	---	---	4 mL	0.020
Water	1120 mL	0.00289	800 mL	0.002
**Total (US$)**		0.46		0.44

[1] Individual cost of each ingredient = [(quantity of the ingredient for 1 kg of diet) × (US$/kg or liter of the ingredient)] ÷ 10^3. * Ingredients to prepare 1 kg of the larval diet proposed previously by Walder et al. [17]: 60 g of wheat germ (US$ 0.103), 3.4 g of agar (US$ 0.20), 60 g of brewer's yeast (US$ 0.08), 60 g of sugar (US$ 0.089), 1 g of sodium benzoate (US$ 0.011), 8 mL of Nipagin solution 0.1% (US$ 0.015), 4 mL of hydrochloric acid (US$ 0.02) and 800 mL of water (US$ 0.002).

Table S2. Nutritional content of the main raw ingredients (according to suppliers' information) and their approximate contribution to larval diets from Embrapa and CENA.

INGREDIENT			NUTRIENTS (g/kg)					
			**Crude protein**	**Total carbohydrates**	**Total Fat**	**Fibers**	**Minerals**	**Vitamins**
Brewer's yeast Brewcell®			503	not declared	2	30	80	not declared
Yellow corn flour Yoki®			72	820	12	38	not declared	not declared
Sugar Caravelas®			0	1,000	0	not declared	0	not declared
DIET	Embrapa	Total (g/kg)	46.8 [1]	276.0 [4]	3.7 [6]	12.9 [8]	4.0 [10]	unknow
		% (*w*/*w*) [2]	4.7	27.6	0.37	1.29	0.4	unknow
	CENA	Total (g/kg)	34.5 [3]	109.2 [5]	0.84 [7]	4.08 [9]	4.8 [11]	unknow
		% (*w*/*w*)	3.5	10.92	0.084	0.41	0.48	unknow

[1] Total estimated protein input (g/kg of diet) = $[(503 \times 50) \div 10^3] + [(72 \times 300) \div 10^3] + 0$. [2] Percentage of the nutrient in 1 kg of diet = (total ingredient input (g/kg) × 100) ÷ 10^3. [3] Total estimated protein input (g/kg of diet) = $[(503 \times 60) \div 10^3] + [(72 \times 60) \div 10^3] + 0$. [4] Total estimated carbohydrate input (g/kg of diet) = $0 + [(820 \times 300) \div 10^3] + [(10^3 \times 30) \div 10^3]$. [5] Total estimated carbohydrate input (g/kg of diet) = $0 + [(820 \times 60) \div 10^3] + [(10^3 \times 60) \div 10^3]$. [6] Total estimated lipid input (g/kg of diet) = $[(2 \times 50) \div 10^3] + [(12 \times 300)$

$\div 10^3$] + 0. [7] Total estimated lipid input (g/kg of diet) = [$(2 \times 60) \div 10^3$] + [$(12 \times 60) \div 10^3$] + 0. [8] Total estimated fiber input (g/kg of diet) = [$(30 \times 50) \div 10^3$] + [$(38 \times 300) \div 10^3$]. [9] Total estimated fiber input (g/kg of diet) = [$(30 \times 60) \div 10^3$] + [$(38 \times 60) \div 10^3$]. [10] Total estimated mineral input (g/kg of diet) = $(80 \times 50) \div 10^3$. [11] Total estimated mineral input (g/kg of diet) = $(80 \times 60) \div 10^3$.
